# Perioperative immunotherapy for hepatocellular carcinoma: adjuvant, neoadjuvant, and biomarker-guided strategies

**DOI:** 10.3389/fmed.2026.1811918

**Published:** 2026-04-10

**Authors:** Kangrui Li

**Affiliations:** Department of Clinical Medicine, Xinjiang Medical University, Urumqi, Xinjiang, China

**Keywords:** adjuvant, biomarkers, circulating tumor DNA, hepatocellular carcinoma, neoadjuvant, perioperative immunotherapy

## Abstract

Hepatocellular carcinoma (HCC) remains difficult to cure after resection or ablation, with high recurrence rates. Perioperative immunotherapy has rapidly evolved, but recent late-phase readouts highlight that benefit is not universal. In IMbrave050, adjuvant atezolizumab plus bevacizumab initially improved recurrence-free survival (RFS), yet longer follow-up suggests attenuation of effect, influencing guideline recommendations. By contrast, adjuvant single-agent checkpoint inhibitor programs have not shown consistent benefit, although selected high-risk subgroups [e.g., microvascular invasion (MVI)] may derive benefit from adjuvant PD-1 blockade in phase II data. Perioperative combinations are emerging, including camrelizumab plus rivoceranib with improved event-free survival, and locoregional-immunotherapy strategies such as transarterial chemoembolization combined with immunotherapy–antiangiogenic regimens. Biomarker-driven selection, circulating tumor DNA for minimal residual disease, resistance-associated alterations (e.g., CTNNB1), and etiology-linked immune phenotypes, will be central to optimizing patient selection and treatment sequencing.

## Graphical Abstract

**Figure:**
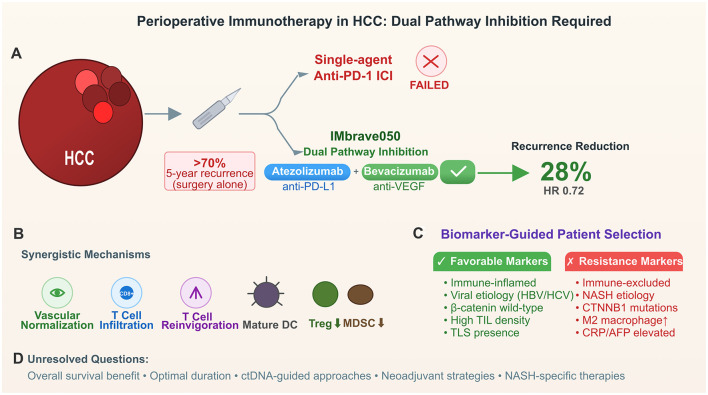
Perioperative immunotherapy in HCC. (A) High recurrence risk following curative resection/ablation. (B) Adjuvant atezolizumab plus bevacizumab reduces recurrence where single-agent ICI failed. (C) Biomarker-guided selection distinguishes responders from resistant disease. (D) Key unresolved questions.

## 1 Introduction

Hepatocellular carcinoma (HCC) ranks as the sixth most common malignancy globally, with approximately 906,000 new cases diagnosed annually and the third leading cause of cancer mortality (1). Although surgical resection and ablation offer potentially curative treatment, more than 70% of patients experience recurrence within 5 years, predominantly within the first 2 years (2, 3). Clinical features including microvascular invasion (MVI), poor differentiation, large tumor size, and multifocal disease consistently predict early relapse. Prior adjuvant strategies uniformly failed: the STORM trial demonstrated no recurrence-free survival (RFS) benefit with sorafenib vs. placebo (median 33.3 vs. 33.7 months) (4), and the phase III PATRON trial likewise did not meet its primary disease-free survival endpoint with adjuvant muparfostat (PI-88) (5).

The landscape shifted with immune checkpoint inhibitors. Although single-agent nivolumab and pembrolizumab showed antitumor activity in early-phase studies, subsequent phase III trials did not meet their prespecified survival endpoints in advanced HCC (5, 6). The breakthrough came with IMbrave150, demonstrating that atezolizumab plus bevacizumab significantly improved survival over sorafenib in advanced disease (median overall survival 19.2 vs. 13.4 months, objective response rate 27.3% vs. 11.9%) (7, 8). Building on this success, ICI-based combinations are being actively evaluated across earlier-stage and embolization-eligible settings, including perioperative and TACE-combination strategies (9).

At present, atezolizumab plus bevacizumab (IMbrave150) and durvalumab plus tremelimumab (HIMALAYA) hold regulatory approval for unresectable or metastatic HCC. No adjuvant, neoadjuvant, or perioperative immune checkpoint inhibitor regimen has yet achieved regulatory approval, and all programmes in these settings remain investigational. Building on the success of these doublets in advanced disease, investigation in the perioperative setting is scientifically compelling: lower tumor burden, a less immunosuppressive microenvironment, and better-preserved hepatic function could further enhance efficacy (10). Against this backdrop, the IMbrave050 trial tested whether adjuvant combination immunotherapy could succeed where prior strategies had failed (11). This review examines current evidence for adjuvant and neoadjuvant immunotherapy, discusses predictive biomarkers including practical constraints on their deployment, and addresses unresolved questions regarding survival benefit, treatment duration, and etiology-specific approaches.

## 2 Adjuvant immunotherapy: clinical evidence and therapeutic advances

### 2.1 The IMbrave050 trial: design, efficacy, and extended follow-up

The IMbrave050 trial was a global phase III study enrolling patients at high recurrence risk following curative resection or ablation (11). High-risk criteria included large tumor size (>5 cm), multifocality, poor differentiation, MVI, or elevated alpha-fetoprotein. Patients were randomized to atezolizumab plus bevacizumab for approximately 12 months or active surveillance. At interim analysis, adjuvant therapy significantly reduced the risk of recurrence or death (*HR* 0.72; 95% *CI* 0.56–0.93; *p* = 0.012), with consistent benefit across prespecified subgroups (11).

However, updated analysis presented at ESMO 2024 with 35.1 months of median follow-up demonstrated substantial attenuation: the hazard ratio deteriorated to 0.90 (95% *CI* 0.72–1.12; *p* not significant), and median RFS numerically favored the surveillance arm (36.0 vs. 33.2 months) (12). Overall survival data remained immature (*HR* 1.26; 95% *CI* 0.85–1.87), numerically favoring surveillance. Whether this convergence reflects delayed recurrence, statistical censoring artifact, or the impact of post-recurrence systemic therapy in the control arm remains uncertain (13). Accordingly, current AASLD and EASL guidance recommends active surveillance or clinical-trial enrolment rather than routine adjuvant systemic therapy (14, 15).

### 2.2 Alternative adjuvant regimens and the importance of dual pathway inhibition

Single-agent checkpoint blockade has not established a consistent adjuvant recurrence-prevention benefit after curative resection or ablation. In KEYNOTE-937, adjuvant pembrolizumab did not improve recurrence-free survival vs. placebo. EMERALD-2 is an ongoing three-arm phase III trial designed to clarify whether bevacizumab adds benefit to adjuvant durvalumab after curative therapy (16). In contrast, the phase III IMbrave050 trial demonstrated improved recurrence-free survival with adjuvant atezolizumab plus bevacizumab vs. active surveillance in patients at high risk of recurrence, suggesting that combination strategies may be required for clinically meaningful perioperative benefit (17).

A notable exception emerged from a Chinese randomized phase II trial evaluating adjuvant sintilimab in 198 patients selected exclusively for MVI (18). Adjuvant sintilimab significantly prolonged RFS compared with active surveillance (median 27.7 vs. 15.5 months; *HR* 0.534; 95% *CI* 0.360–0.792; *p* = 0.002), with grade 3–4 adverse events in only 12.4% of patients (18). The enrolled population was predominantly HBV-associated, and phase III validation is required before any practice change. The EMERALD-2 trial addresses the contribution of anti-VEGF combination directly through a three-arm design comparing durvalumab monotherapy, durvalumab plus bevacizumab, and placebo; preliminary results confirmed that durvalumab alone did not improve RFS, while combination arm data remain pending (16).

**Figure 1:**
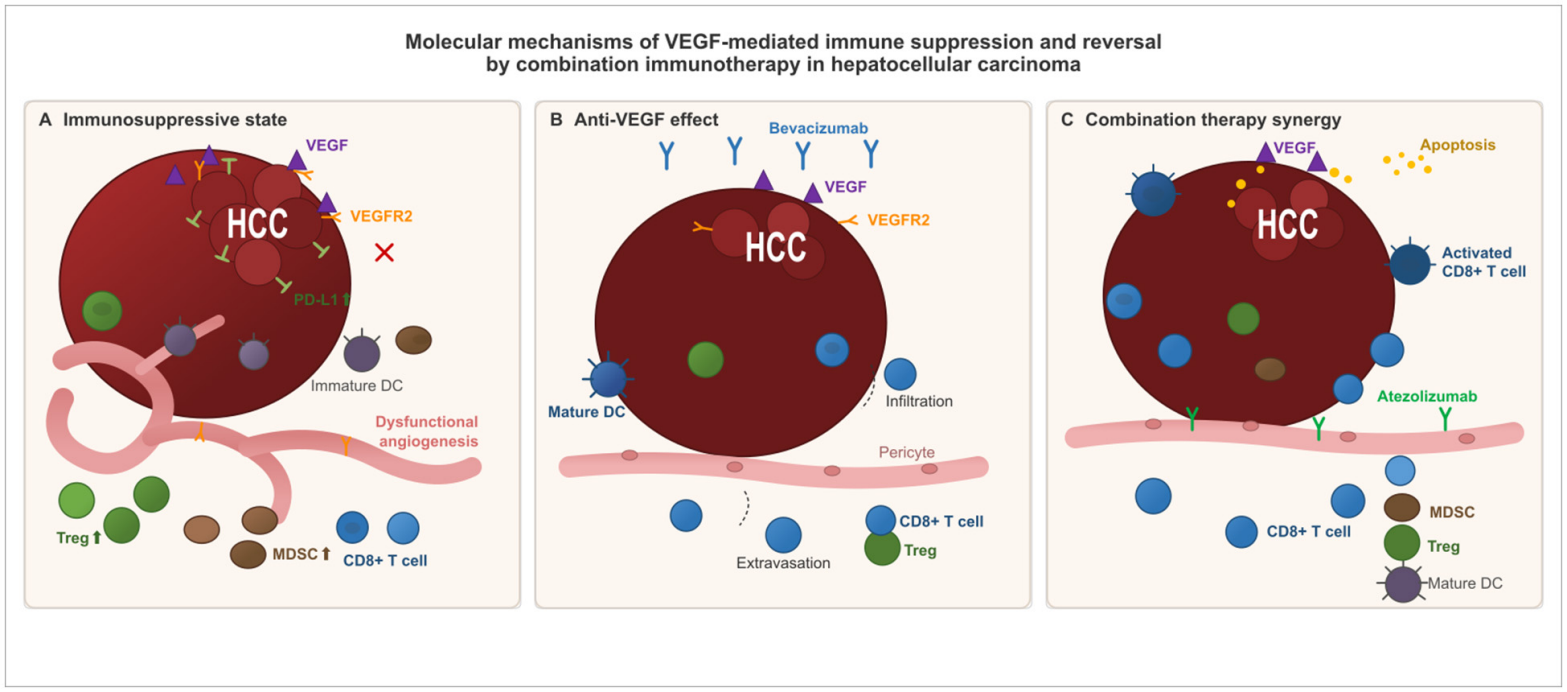
VEGF-mediated immune suppression and reversal by combination immunotherapy in HCC. **(A)** VEGF promotes dysfunctional angiogenesis, inhibits dendritic cell (DC) maturation, upregulates PD-L1, and impairs CD8+ T cell infiltration while facilitating Treg and MDSC accumulation. **(B)** Anti-VEGF therapy (bevacizumab) normalizes vasculature, enhancing T cell extravasation and DC function. **(C)** Concurrent PD-L1 blockade (atezolizumab) reinvigorates exhausted T cells. Dual pathway inhibition overcomes immunosuppression where single-agent therapy fails.

The rationale for combination therapy rests on complementary immunological mechanisms. VEGF promotes dysfunctional tumor vasculature, impairs DC maturation, and fosters accumulation of Tregs and MDSCs. Anti-VEGF therapy normalizes vasculature and restores DC function, thereby potentiating checkpoint blockade (19). These converging mechanisms suggest that simultaneous inhibition of both angiogenic and immune checkpoint pathways may be a prerequisite for effective adjuvant immunotherapy in HCC (Figure 1). All major trials are summarized in Table 1.

**Table 1:** Summary of key immunotherapy trials in the perioperative and locoregional-combination settings for HCC.

Trial and setting	Regimen; *N*; enrolment region	Key outcomes (*HR* [95% *CI*]; median survival)	Status and notes
**IMbrave050** adjuvant	Atezo + Bev vs. surveillance *N* = 668; global	Interim: *HR* 0.72 (0.56–0.93); *p* = 0.012Updated: *HR* 0.90 (0.72–1.12); *p* = NSmRFS 33.2 vs. 36.0 mo OS HR 1.26 (0.85-1.87); immature	Initially positive; signal attenuated at 35-mo follow-up; OS numerically favors surveillance; not guideline-endorsed (12, 15)
**KEYNOTE-937** adjuvant	Pembrolizumab vs. placebo*N* = 959; global	HR NR; *p* = NS mRFS 46.7 vs. 45.5 mo	Negative (16)
**CheckMate-9DX** adjuvant	Nivolumab vs. placebo *N* = 545; Global	HR NR; *p* = NS	Negative (16)
**EMERALD-2** adjuvant	Durva ± Bev vs. placebo *N* = 908; global	Pending	Ongoing; 3-arm design (17)
**Sintilimab Ph II** adjuvant (MVI only)	Sintilimab vs. surveillance *N* = 198; China (HBV)	*HR* 0.534 (0.360–0.792); *p* = 0.002 mRFS 27.7 vs. 15.5 mo	Positive (Ph II); MVI required for enrolment; HBV-predominant; Ph III validation needed (18)
**JUPITER-04** adjuvant	Toripalimab vs. placebo	NR	Ongoing (16)
	*N* = 402; China		
**CARES-009** perioperative	Camrelizumab + rivoceranib vs. surgery alone *N* = 294; China (HBV)	*HR* 0.59 (0.41–0.85); *p* = 0.004 mEFS 42.1 vs. 19.4 mo	Positive (Ph 2/3); G ≥ 3 AEs 38%; HBV-predominant; OS pending (27)
**Early-phase neoadjuvant studies** neoadjuvant	Cabozantinib + nivolumab (*n* = 15); nivolumab ± ipilimumab (*n* ~ 30); cemiplimab (*n* = 21), USA	R0 resection 80%; MPR 42% (23)	Ph I/II proof-of-concept; TLS enrichment in responders; no HR data available across studies (23–26)
		MPR 20% (26)	
		pCR in subset (22)	
**EMERALD-1** TACE + systemic (intermediate HCC)	TACE + Durva + Bev vs. TACE + placebo	HR 0.77 (0.61–0.98); p = 0.032 mPFS 15.0 vs. 8.2 mo ORR 43.6% vs. 29.6%	Positive; first positive Ph III TACE trial in >20 years; TACE + Durva alone: *HR* 0.94, no benefit (28)
**LEAP-012** TACE + systemic (intermediate HCC)	TACE + Lenva + pembro vs. TACE + placebo *N* = 480; global (Asia-predominant) mPFS 14.6 vs. 10.0 mo ORR 46.8% vs. 33.3%	*HR* 0.66 (0.51–0.84); *p* = 0.0002	PFS benefit but OS futility (*HR* 0.80; *p* = 0.087); G3–4 AEs 71.3%; 4 treatment-related deaths; closed Oct 2025 (29)

Bold text indicates Trial name (clinical trial identifier). AEs, adverse events; Atezo, atezolizumab; Bev, bevacizumab; Durva, durvalumab; EFS, event-free survival; FU, follow-up; G, grade; HBV, hepatitis B virus; Lenva, lenvatinib; mEFS**/**mPFS**/**mRFS, median EFS/PFS/RFS; MPR, major pathological response; MVI, microvascular invasion; N, number of patients; NR, not reported; NS, not significant; ORR, objective response rate; OS, overall survival; Pembro, pembrolizumab; pCR, pathological complete response; PFS, progression-free survival; Ph, phase; RFS, recurrence-free survival; TACE, transarterial chemoembolisation; TLS, tertiary lymphoid structures.

### 2.3 Optimizing treatment duration and dosing strategies

The 12-month treatment course in IMbrave050 was selected empirically, and the post-cessation convergence of RFS curves raises unresolved questions about whether benefit reflects elimination of micrometastatic disease or a transient cytostatic effect. In clinical practice, treatment duration will likely require individualization guided by early response biomarkers, analogous to the use of pathological complete response in KEYNOTE-522 to tailor adjuvant therapy in triple-negative breast cancer (20).

The feasibility of intermittent or finite-duration immunotherapy also merits exploration. Melanoma data demonstrate durable disease control in some patients following treatment discontinuation, suggesting development of immunological memory (21). Circulating tumor DNA (ctDNA) kinetics or minimal residual disease (MRD) status could identify patients suitable for abbreviated courses while flagging those with persistent high-risk features for extended therapy. Given the substantial costs of ICI-antiangiogenic regimens in advanced HCC (22), biomarker-guided escalation and de-escalation is preferable to a fixed course applied uniformly to all patients.

## 3 Neoadjuvant approaches and conversion therapy strategies

### 3.1 Biological rationale and pathologic response patterns

Neoadjuvant immunotherapy offers the theoretical advantage of treating an intact tumor, exposing the immune system to the full neoantigen repertoire and enabling real-time pathological response assessment to guide subsequent management (23). The proof-of-concept study by Ho et al. evaluated neoadjuvant cabozantinib plus nivolumab in 15 patients with locally advanced HCC; 80% underwent successful R0 resection, with major pathological response in 42%, and spatial transcriptomics demonstrated tertiary lymphoid structure (TLS) formation in responders (23). Further proof-of-concept signals have been provided by perioperative nivolumab with or without ipilimumab, neoadjuvant ipilimumab plus nivolumab in the PRIME-HCC study, and neoadjuvant cemiplimab (24–26).

The CARES-009 trial provides the strongest randomized evidence supporting perioperative immunotherapy to date. This phase 2/3 study randomized 294 patients with resectable, intermediate-to-high-risk HCC (CNLC stages Ib-IIIa) to perioperative camrelizumab plus rivoceranib vs. surgery alone (27). At a median follow-up of 33.1 months, the perioperative combination significantly improved event-free survival (median 42.1 vs. 19.4 months; *HR* 0.59; 95% *CI* 0.41–0.85; *p* = 0.0040), without compromising surgical feasibility, though grade 3 or higher adverse events occurred in 38% of the perioperative group (27). Conducted exclusively in China with a predominantly HBV-associated population, these results warrant caution when extrapolating to non-Asian or non-viral HCC cohorts.

### 3.2 Integration with locoregional therapies: the EMERALD and LEAP experience

TACE induces tumor necrosis, releases antigens that may prime systemic immunity, and simultaneously creates hypoxia that upregulates VEGF expression, providing a biological rationale for combining TACE with dual ICI-antiangiogenic blockade. The EMERALD-1 trial represents a landmark achievement as the first positive phase III TACE combination trial in over 2 decades. This study randomized 616 patients with intermediate-stage HCC to TACE plus durvalumab and bevacizumab, TACE plus durvalumab, or TACE plus placebo. The triplet combination demonstrated significant PFS improvement: median 15.0 vs. 8.2 months (*HR* 0.77, 95% *CI* 0.61–0.98; *p* = 0.032), with objective response rate of 43.6% vs. 29.6%. Importantly, durvalumab monotherapy with TACE showed no benefit over placebo (*HR* 0.94; *p* = 0.64), reinforcing the necessity of dual immunotherapy-antiangiogenic blockade. Grade 3–4 treatment-related adverse events occurred in 32.5%, with no treatment-related deaths (28).

The LEAP-012 trial randomized 480 patients with intermediate-stage HCC to TACE combined with lenvatinib plus pembrolizumab, or TACE with dual placebo (29). Although significant PFS improvement was achieved (median 14.6 vs. 10.0 months; *HR* 0.66; 95% *CI* 0.51–0.84; *p* = 0.0002; ORR 46.8%), the trial was closed in October 2025 following a prespecified OS futility analysis (interim *HR* 0.80; *p* = 0.087) (29). The substantial toxicity burden, with grade 3–4 adverse events in 71.3% of patients and four treatment-related deaths, underscores that PFS benefit alone is insufficient to justify a regimen with this toxicity profile, and that overall survival remains the definitive endpoint in this setting.

### 3.3 Conversion therapy: expanding surgical candidacy

Conversion therapy in the HCC context refers to the use of systemic and/or locoregional treatment with the specific intent of rendering initially unresectable disease surgically resectable, as determined by formal multidisciplinary team reassessment. This goal is distinct from downstaging in the liver transplant context, which aims to reduce tumor burden within morphological transplant eligibility criteria. Resectability reassessment is often performed after approximately 2–4 treatment cycles, depending on the regimen and treatment response, and usually integrates three domains of consideration: anatomical (anticipated future liver remnant volume of at least 30%, or at least 40% in the setting of underlying cirrhosis, along with the relationship of the tumor to major portal and hepatic vein branches), functional (ALBI grade, serum bilirubin, platelet count), and oncological (disappearance of extrahepatic metastases, AFP trajectory, and absence of progressive macrovascular invasion).

Available evidence for conversion therapy remains heterogeneous and should be interpreted cautiously. In a retrospective Japanese study of atezolizumab plus bevacizumab in 156 Child-Pugh A patients with unresectable HCC, 17 of 156 patients (10.9%) became eligible for conversion therapy after tumor response, and curative conversion with surgical resection or radiofrequency ablation was associated with longer recurrence-free survival than TACE or treatment discontinuation (30). Correlative analyses further suggest that baseline immune and molecular features may influence sensitivity to atezolizumab plus bevacizumab, and thus may partly shape the likelihood of achieving a conversion window, although such biomarkers are not yet ready for routine surgical selection (31).

HAIC-based strategies may be particularly relevant for patients with bulky intrahepatic disease or macrovascular invasion. In a phase III trial in unresectable large HCC, FOLFOX-HAIC achieved a higher response rate than TACE (46% vs. 18%) and improved both progression-free and overall survival (32). In the phase III FOHAIC-1 trial, HAIC-FO resulted in tumor downstaging in 16 of 130 patients (12.3%), including 15 who subsequently underwent curative surgery or ablation (33). However, prospective randomized trials specifically designed for conversion therapy remain lacking; future studies should incorporate standardized resectability criteria, prespecified reassessment schedules, and R0 resection rate as a primary endpoint.

## 4 Predictive biomarkers and personalized treatment selection

### 4.1 Tumor microenvironment classification and immune phenotypes

Heterogeneous immunotherapy responses in HCC highlight the need for predictive biomarkers. Unlike malignancies where PD-L1 expression or microsatellite instability reliably inform treatment, HCC has proven more complex. Traditional markers including PD-L1 expression and tumor mutational burden (TMB) demonstrate limited predictive value in HCC (34). In correlative analyses of atezolizumab plus bevacizumab, TMB has not emerged as a clinically useful treatment-selection marker (34).

Spatial transcriptomics and single-cell sequencing have defined three tumor immune phenotypes with distinct prognostic implications (35). Immune-inflamed tumors exhibit dense CD8+ T cell infiltration, interferon-gamma signatures, and active antigen presentation, conferring checkpoint inhibitor responsiveness. Immune-excluded tumors display T cells restricted to tumor margins, often due to stromal barriers or molecular exclusion signals. Immune-desert tumors lack meaningful T cell infiltration, reflecting failed immune priming.

Wnt/beta-catenin pathway activation, present in 11–37% of HCC cases via CTNNB1 mutations or related alterations, represents the most robustly validated mechanism of primary immunotherapy resistance. In correlative analyses, patients with CTNNB1-mutant tumors demonstrated a 0% objective response rate and 100% progressive disease on ICI therapy, with significantly shortened median survival compared with wild-type (9.1 vs. 15.2 months) (34, 36). These mutations drive immune exclusion through impaired DC recruitment and defective T cell priming. Strategies to therapeutically overcome Wnt-mediated resistance remain under active investigation.

### 4.2 Gene expression signatures and molecular classifiers

**Figure 2:**
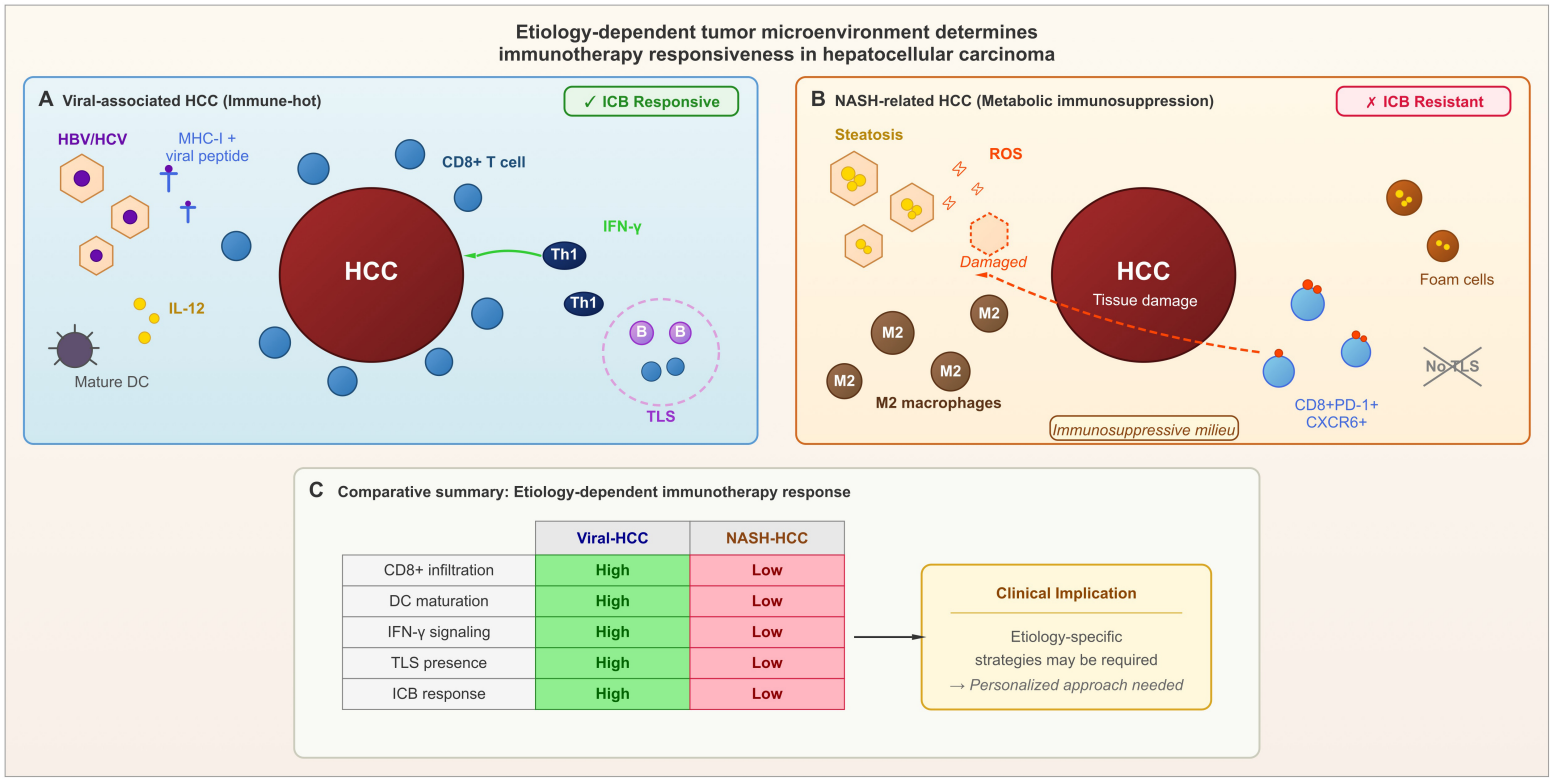
Etiology-dependent tumor microenvironment determines immunotherapy responsiveness in HCC. **(A)** Viral HCC (HBV/HCV) develops amid chronic immune activation with mature DCs, Th1 polarization, IFN-γ signaling, CD8+ T cell infiltration, and tertiary lymphoid structures (TLS), conferring ICB responsiveness. **(B)** NASH-related HCC arises from metabolic dysfunction, featuring M2 macrophages, dysfunctional CD8+ T cells, and scarce TLS, leading to ICB resistance. **(C)** Comparative summary underscores the need for etiology-specific immunotherapy strategies.

Correlative studies of atezolizumab plus bevacizumab suggest that benefit is enriched in tumors with T-effector/immune-active programs or angiogenesis-related signatures, whereas resistance is associated with immune-excluded or myeloid-rich states (34). Tumor etiology also influences responsiveness. Virus-associated HCC (HBV/HCV) tends to be immunologically hot, featuring mature DCs, IFN-gamma signaling, and TLS, whereas MAFLD/NASH-related HCC exhibits an immune-exhausted microenvironment with impaired immune surveillance (37) (Figure 2). Preclinical models showed that NASH-driven HCCs are less responsive to checkpoint blockade due to impaired immune surveillance. However, current clinical evidence does not support withholding immunotherapy solely on the basis of non-viral etiology (38, 39). Given, however, that CARES-009 and the sintilimab phase II study enrolled predominantly HBV-associated patients (18, 27), their results may not translate directly to Western populations where MAFLD is the dominant etiology. Meta-analyses of viral hepatitis-associated HCC suggest that etiology may modulate response and survival outcomes, although between-study heterogeneity remains substantial (39).

### 4.3 Circulating biomarkers and liquid biopsy

ctDNA has emerged as a promising tool for MRD detection and prognostication in perioperative HCC. A meta-analysis of 10 studies encompassing 793 patients demonstrated that postoperative ctDNA positivity strongly predicts adverse outcomes, with a pooled hazard ratio of approximately 4.5 for overall or disease-free survival (40). Tumor-informed serial assays can detect ctDNA after curative-intent therapy and identify patients at markedly increased risk of recurrence, often before conventional radiological confirmation (41).

Beyond ctDNA, the CRAFITY score and the ALBI grade are accessible blood-based markers with prognostic value in patients with HCC treated with immune checkpoint inhibitors (42, 43). Patients with lower CRAFITY scores generally experience better outcomes than those with higher scores, whereas worse ALBI grades identify patients with poorer liver reserve and inferior survival. Both scores are derivable from routine blood tests, making them accessible bridging biomarkers in a broad range of clinical settings.

### 4.4 Practical constraints: cost, standardization, and global accessibility

Despite their scientific promise, most advanced biomarker platforms face formidable barriers to clinical implementation. Tumor-informed ctDNA assays remain costly, require specialized sequencing infrastructure, and are not yet standardized across platforms in HCC (41, 44). There is no consensus on optimal assay design, variant allele fraction thresholds, or the operational definition of MRD positivity, making cross-study comparison difficult (44). For resource-constrained settings, the CRAFITY score and the ALBI grade provide accessible alternatives derivable from routine laboratory results (42, 43). Clinicians and trialists should recognize that the optimal biomarker strategy may differ substantially between a well-resourced academic centre and a community hospital in an HBV-endemic region.

## 5 Future outlook: trials on the horizon

Multiple ongoing trials will further inform perioperative immunotherapy strategies. In the adjuvant setting, EMERALD-2 (durvalumab ± bevacizumab) will determine whether anti-VEGF augmentation improves outcomes after resection, and additional peri-curative strategies are under exploration, including adjuvant toripalimab (JUPITER-04; NCT03859128) and LRT-combined regimens such as AB-LATE02 (NCT04727307; RFA-based) and NIVOLEP (NCT03630640; electroporation-based). In the neoadjuvant space, KEYNOTE-934 and ORIENT-32B are assessing perioperative pembrolizumab-based and sintilimab-based combinations, respectively. Early-phase GPC3-targeted CAR-T cell therapy has also shown feasibility and preliminary activity in advanced HCC (45). Notably, the CheckMate 9DW trial demonstrated that nivolumab plus ipilimumab improved overall survival over lenvatinib or sorafenib as first-line therapy in advanced HCC (median OS 23.7 vs. 20.6 months; *HR* 0.79; 95% *CI* 0.65–0.96; *p* = 0.018; ORR 36%; median response duration 30.4 months), receiving FDA approval in April 2025, providing a direct rationale for investigating dual checkpoint blockade in perioperative contexts (46).

Novel immune targets remain of interest but require confirmatory testing. The phase III IMbrave152/SKYSCRAPER-14 study is evaluating whether tiragolumab can improve outcomes when added to atezolizumab plus bevacizumab, and the randomized MORPHEUS-Liver study suggested that this anti-TIGIT strategy may increase activity without substantially worsening safety (47, 48). Personalized neoantigen vaccination represents a promising frontier: in a phase 1/2 study, the GNOS-PV02 personalized neoantigen DNA vaccine combined with pembrolizumab achieved an objective response rate of 30.6% and a median overall survival of 19.9 months in TKI-pretreated advanced HCC (49). These results appear encouraging relative to historical experience with pembrolizumab monotherapy, while acknowledging the limitations of cross-trial comparisons (6). Post-operative ctDNA-guided therapy, escalating treatment for MRD-positive patients and de-escalating for MRD-negative patients, mirrors evolving paradigms in colorectal and breast cancer and represents an appealing direction for adaptive perioperative trial design.

## 6 Conclusions

Perioperative immunotherapy for HCC is rapidly maturing, yet recent late-phase data indicate that benefit is regimen- and population-dependent rather than universal. The adjuvant atezolizumab–bevacizumab signal in IMbrave050 appears to attenuate with longer follow-up, and adjuvant single-agent PD-1/PD-L1 programs have not shown consistent recurrence-prevention benefit. In contrast, perioperative combination strategies (e.g., camrelizumab plus rivoceranib) and selected high-risk subgroups (e.g., microvascular invasion) suggest a path forward. Future progress will rely on trial-based adoption and biomarker-driven tailoring, integrating tissue contexture, blood-based markers, and ctDNA-defined MRD, with attention to etiology-specific immunobiology.
